# Stressed! Grab a bite? Stress eating in adults with Attention-Deficit/Hyperactivity Disorder: An Ecological Momentary Assessment study

**DOI:** 10.1016/j.nsa.2025.105509

**Published:** 2025-02-08

**Authors:** Alea Ruf, Andreas B. Neubauer, Elena D. Koch, Ulrich Ebner-Priemer, Andreas Reif, Silke Matura

**Affiliations:** a Goethe University Frankfurt, University Hospital, Department of Psychiatry, Psychosomatic Medicine and Psychotherapy, Germany; b DIPF | Leibniz Institute for Research and Information in Education, Frankfurt am Main, Germany; c Center for Research on Individual Development and Adaptive Education of Children at Risk (IDeA), Frankfurt am Main, Germany; d Mental mHealth Lab, Institute of Sports and Sports Science, Karlsruhe Institute of Technology (KIT), Karlsruhe, Germany; e Department of Psychiatry and Psychotherapy, Central Institute of Mental Health, Medical Faculty Mannheim, Heidelberg University, Mannheim, Germany; f Fraunhofer Institute for Translational Medicine and Pharmacology ITMP, Theodor-Stern-Kai 7, 60596, Frankfurt am Main, Germany

**Keywords:** Stress eating, Adult ADHD, Ecological momentary assessment, Trait and state impulsivity, Food intake

## Abstract

Meta-analytical evidence suggests that adults with Attention-Deficit/Hyperactivity Disorder (ADHD) face a 70% higher risk for obesity. Elevated levels of stress, the lack of adequate stress coping strategies, and the tendency to overeat might make individuals with ADHD vulnerable to stress-induced eating, i.e., engaging in (over)eating when feeling stressed – a behavioural pathway through which ADHD symptomatology may contribute to obesity. Research indicates that particularly impulsivity symptoms of ADHD are associated with overeating. This study is the first to use Ecological Momentary Assessment (EMA) to assess (1) whether stress is generally associated with (over)eating in adults with ADHD and (2) whether trait and state impulsivity moderate the stress and eating relationship. Thirty-six adults with ADHD completed a 3-day EMA period. Participants reported perceived stress and state impulsivity eight times a day (signal-contingent) and recorded food intake (event-contingent). Multilevel two-part models were used to study the relationship between stress and the occurrence as well as the amount of food intake. Stress was not related to the occurrence and the amount of food intake. Trait and state impulsivity did not moderate the stress and eating relationship. This study provides preliminary evidence that adults with ADHD might not be at particular risk for stress eating. Future studies are needed to replicate these findings. Advancing our understanding of eating – a central, indispensable human behaviour – in this under-researched at-risk population is crucial given its significant public health impact due to the high disease burden and personal suffering associated with obesity and ADHD.

## 1 Introduction

The global prevalence of obesity has not only tripled since 1975 (World Health Organization), but estimates predict further increases in global levels of overweight and obesity from 38% in 2020 to over 50% in 2035 (World Obesity Federation, 2023). These trends are alarming as obesity is linked to numerous negative health outcomes, such as cancer and diabetes, but also mental disorders, like depression (for an overview see World Health Organization. Regional Office for Europe, 2022). Considerable evidence has shown that there is a strong link between obesity and Attention-Deficit/Hyperactivity Disorder (ADHD) in adults (see Cortese, 2019 for an overview). Originally conceptualized as a neurodevelopmental condition in childhood, it is now recognized that ADHD persists into adulthood, with around 2.5% of adults reporting clinically-significant symptoms of inattention, hyperactivity, and impulsivity (Fayyad et al., 2017; Simon et al., 2009). Meta-analytical evidence suggesting that adults with ADHD are 70% more likely to be affected by obesity (Cortese et al., 2015) highlights the critical need to better understand mechanisms underlying this association. Genetic factors, dopaminergic dysfunction, fetal programming, and inflammatory processes are discussed as biological mechanisms underlying the associations between ADHD and obesity (for overviews see Cortese, 2019; Cortese et al., 2015; Hanć and Cortese, 2018).

A behavioural pathway through which ADHD psychopathology may contribute to obesity is stress-induced eating: A systematic review not only found a positive association between ADHD and disordered eating, but also that particularly impulsivity symptoms of ADHD are positively associated with overeating (Kaisari et al., 2017). Beyond that, individuals with ADHD show emotion regulation deficits (Beheshti et al., 2020; Christiansen et al., 2019) and maladaptive stress coping strategies (Barra et al., 2021), while at the same time reporting elevated levels of perceived stress (Combs et al., 2012; Hirvikoski et al., 2009; Lackschewitz et al., 2008). Taken together, these findings suggest that adults with ADHD might be at particular risk for stress-induced eating (i.e., [over]eating as a response to stress), as they seem to (1) experience more stress, (2) lack adequate strategies to cope with stress, and (3) show a tendency to overeat. Hence, adults with ADHD might engage in (over)eating to cope with stress which may contribute to overweight and obesity in the long term. Even though adults with ADHD may particularly benefit from measures targeting stress in order to prevent or reduce overweight and obesity, the number of studies assessing the relationship between stress and eating in adults with ADHD is limited.

Research on stress eating in healthy adults has shown that individuals seem to differ in the dietary response to stress, as such that some eat more, some eat less, while others show no change (Torres and Nowson, 2007; Araiza and Lobel, 2018; Hill et al., 2021). The individual-difference model of stress-induced eating proposes that the dietary response to stress is a trait-like characteristic which is determined by differences in attitudes, learning history, or biology (Greeno and Wing, 1994). This model emphasizes the role of person-specific traits and experiences in determining an individual's dietary response to stress. Even though research has tried for decades to identify person-characteristics that explain individual differences in the dietary response to stress, findings remain highly inconclusive. Beyond that, first evidence indicates that an individual's dietary response to stress might not be as stable as yet assumed (Ruf et al., 2023). Instead, individuals might not always engage in the same dietary response to stress as time-varying factors might shape their response. Based on this, Ruf et al. (2023) proposed an expansion of the individual-difference model, the dynamic individual-difference model, which accounts for time-varying factors that might alter an individual's dominant dietary response to stress. According to the expanded model, it appears important not only to identify individuals at risk for stress-induced eating, but also to identify situations with an elevated risk for stress-induced eating.

The aim of the present study was to use Ecological Momentary Assessment (EMA) to assess the relationship between stress and actual food intake in the daily life of adults with ADHD. Actual food intake refers to capturing all consumed foods and drinks as well as their amounts, which can then be used to generate nutritional values (e.g., energy intake in kilocalories [kcal]). Three research questions were addressed: (1) It was assessed whether stress is generally associated with (increased) food intake in adults with ADHD. This would imply that all individuals with ADHD are at risk for stress-induced eating. (2) Based on the individual-difference model, it was assessed whether trait impulsivity – an aspect of ADHD psychopathology particularly associated with overeating (Kaisari et al., 2017) – moderates the stress and eating relationship. This could mean that only individuals with ADHD who show higher levels of trait impulsivity are at risk for stress eating. (3) ADHD symptoms are dynamic in nature (Koch et al., 2021). Given that impulsivity not only fluctuates from day to day, but also within days (Griffin et al., 2020), particularly in adults with a history of ADHD (Pedersen et al., 2019), it was assessed whether state impulsivity moderates the stress and eating relationship based on the dynamic individual-difference model. This could suggest that individuals with ADHD are at risk for stress eating only in situations in which they are more impulsive. Hence, the aim for the present study is to take first steps towards a better understanding of the interplay of stress, impulsivity, and eating behaviour in adults with ADHD by collecting detailed, longitudinal data in daily life.

## 2 Methods

### 2.1 Procedure

Data of the present study were collected as part of the APPetite study which comprised two in-person sessions and a three-day EMA period (consecutive days: two weekdays and one weekend day). In the first in-person session, participants provided sociodemographic information, completed questionnaires, and received detailed training on how to use the EMA tool, the APPetite-mobile-app (see Ruf et al., 2021a for a detailed description of the APPetite study and the APPetite-mobile-app). Beyond that, body weight and height were measured to calculate participants' BMI. The EMA protocol of the APPetite-mobile-app comprises eight semi-random signal-contingent prompts per day between 8 a.m. and 10 p.m. which capture perceived stress, state impulsivity, and food availability. Semi-random prompts (i.e., prompts spread out randomly with the prerequisite to have at least 1 h between prompts) were chosen so the assessed situations better reflect participants’ real life, as participants did not know the exact time of the next prompt and could therefore not prepare for it (e.g., stop what they are doing right before the prompt). The incorporated food record assesses actual food intake event-contingently, i.e., participants were asked to record what they eat and drink as soon as possible after consuming it. Additionally, a time-contingent prompt at 9 p.m. asks participants to indicate whether all consumed foods and drinks of the day are recorded and to record any missing foods and drinks. The local ethics committee of the faculty of medicine of the Goethe University Frankfurt (Ethikkommission des Fachbereichs Medizin der Goethe-Universität) approved the study (reference number: 192/18). All participants declared that they understood the study procedure and signed a written informed consent.

### 2.2 Sample

Adults with ADHD who previously participated in one of three studies at the University Hospital in Frankfurt were invited to take part in the APPetite study: (1) the PROUD study (Mayer et al., 2018), (2) the BipoLife-A1 study (Pfennig et al., 2020; Ritter et al., 2016), and (3) the PROBIA study (Arteaga-Henríquez et al., 2020). The inclusion and exclusion criteria of the three studies can be found in Supplement C. Only participants of at least 18 years of age were invited by email and phone to take part in the APPetite study. In total, 43 adults with ADHD agreed to participate in the APPetite study. Only data collected as part of the APPetite study were used in the present paper. Data from the PROUD, BipoLife-A1, and PROBIA study were not used. Note that data reported in Ruf et al. (2023) were also collected as part of the APPetite study, but only included participants without ADHD. Hence, the sample of Ruf et al. (2023) and the current sample are distinct samples and do not overlap. Data of seven participants were excluded due to a low number of completed prompts (10 or below) and/or incomplete records of food intake (e.g., only one meal recorded). The final sample included 36 participants (see sociodemographic information in Table 1).

**Table 1:** Sociodemographic characteristics of the final sample (*N* = 36).

Variable	*n*/*M*	%/*SD*
Gender		
Female	20	55.56%
Male	16	44.44%
Age (years)	35.64	12.48
Marital status		
Single	15	41.67%
In a relationship	12	33.33%
Married	6	16.67%
Divorced	3	8.33%
Highest level of education		
Certificate of Secondary Education	6	16.67%
School-leaving examination (*Abitur*)	8	22.22%
Completed vocational training	7	19.44%
University degree	15	41.67%
Monthly gross income		
0 to 1000 €	14	38.89%
1001 to 2000 €	10	27.78%
2001 to 3000 €	7	19.44%
3001 to 4000 €	3	8.33%
4001 to 5000 €	0	0%
Over 5000 €	2	5.56%
Nationality		
German only	29	80.56%
German and other	5	13.89%
Other only	2	5.56%
BMI	29.18	7.8
Weight Status		
Underweight (BMI <18.5)	1	2.78%
Normal weight (18.5 ≤ BMI <25)	11	30.56%
Overweight (25 ≤ BMI <30)	13	36.11%
Obesity (BMI ≥30)	11	30.56%
ADHD Medication		
Yes	20	55.56%
No	16	44.44%
Comorbidities		
Depression	14	38.89%
Borderline personality disorder	5	13.89%
Eating disorder	0	0%
Depression score^a^	12.14	12.51

a Beck Depression Inventory-II.

### 2.3 Measures

#### 2.3.1 Food intake

The food record of the APPetite-mobile-app (Ruf et al., 2021a) was used to capture actual food intake following a 6-step process: (1) selection of meal type, (2) entry of time of intake, (3) selection of consumed foods and drinks, (4) specification of consumed amounts, (5) presentation of reminder for commonly forgotten foods, and (6) indication of predominant reason for eating or drinking. The collected food recordings were transferred to myfood24-Germany (Koch et al., 2020) by trained staff to generate nutritional values (i.e., energy intake in kcal). Findings of a feasibility, usability, and validation study suggest that the APPetite-mobile-app is a feasible and valid tool to assess actual food intake (Ruf et al., 2021a).

#### 2.3.2 Food availability

Each EMA prompt asked participants to rate food availability since the last prompt (since waking up in the first prompt of each day) on a visual analogue scale from 0 (*not available at all*) to 100 (*easily available*). These ratings were used to exclude time intervals in which food was unavailable (see section 2.4), since the effect of stress on food intake can only be studied meaningfully when participants have access to food.

#### 2.3.3 Perceived stress

Perceived stress was assessed based on three items which were adapted from Reichenberger et al. (2018). The first item asked participants to rate how stressed they felt since the last prompt on a visual analogue scale from 0% (*not at all*) to 100% (*very stressed*). Based on the Perceived Stress Scale (Cohen et al., 1983), two stress items assessed whether participants felt that they “could not cope with all the things they had to do” and whether they were “on top of things” since the last prompt on a visual analogue scale from 0 (*not at all*) to 100 (*very much*). Participants were asked to rate stress *since waking up* instead of *since the last prompt* in the first prompt per day. An average stress score was calculated for each prompt based on the three items (third item reversed). In the final dataset, McDonald's Omegas (Geldhof et al., 2014) were 0.671 (within) and 0.917 (between) for the stress items.

#### 2.3.4 State impulsivity

The Momentary Impulsivity Scale (MIS; Tomko et al., 2014) was used to capture state impulsivity. The MIS has been shown to provide information regarding within-individual variability in impulsivity across and within days (Tomko et al., 2014). Each of the four items of the MIS comprises a statement (e.g., “I said things without thinking”). Participants reported on a 5-point scale how well each statement describes their behaviour, cognition, and experiences since the last prompt or since waking up in the first daily prompt. The four ratings are summed up to an overall impulsivity score (higher values indicating greater state impulsivity). To improve differentiability across response options, the original response scale (1 = *very slightly or not at all*; 2 = *a little*; 3 = *moderately*; 4 = *quite a bit*; 5 = *extremely*) was slightly altered during translation (1 = *nicht zutreffend*, 2 = *eher nicht zutreffend*, 3 = *teils-teils*, 4 = *eher zutreffend*, 5 = *zutreffend*). McDonald's Omega of the MIS was 0.561 (within) and 0.841 (between) in the final dataset.

#### 2.3.5 Trait impulsivity

Trait impulsivity was assessed with the 59-item UPPS-P Impulsive Behavior Scale (Urgency Premeditation Perseverance and Sensation Seeking Impulsive Behavior Scale; Lynam et al., 2006). Each item contains a statement (e.g., “It is hard for me to resist acting on my feelings“). Participants rated how strongly they (dis)agree with each statement on a 4-point-scale from *agree strongly* to *disagree strongly*. The UPPS-P captures impulsivity as a multifaceted construct which includes the following subscales: negative urgency (12 items), positive urgency (14 items), (lack of) premeditation (11 items), (lack of) perseverance (10 items), and sensation seeking (12 items). The German translation by Schmidt et al. (2008) of the subscales negative urgency, (lack of) premeditation, (lack of) perseverance, and sensation seeking were used. The translate-back-translate-procedure was applied to translate the English items of the positive urgency subscale to German. Item ratings of each subscale were summed up and a total impulsivity score was calculated (mean of the subscales). Internal consistency was α = .92 for negative urgency, α = .81 for premeditation, α = .82 for perseverance, α = .89 for sensation seeking, and α = .93 for positive urgency in the final sample.

### 2.4 Data preprocessing

Out of the 864 scheduled EMA prompts (=24 prompts for each of the 36 participants), two prompts were not delivered (likely due to the smartphone being switched off), 15 dismissed, 60 ignored, and 15 incomplete. The mean compliance (complete prompts relative to received prompts) was 89.6% (*SD* = 10.5).

Since stress and state impulsivity were rated for a time interval (i.e., time between current prompt and previous prompt/waking up), each of the stress and state impulsivity assessments (predictors) were paired with concurrent energy intake in kcal (outcome), i.e., the sum of any energy intake within the respective time interval. Due to the semi-random sampling protocol, the assessment of stress ‘since waking up’ in the first prompt, and the option to postpone prompts, the length of the time intervals varied considerably. Therefore, time intervals which were shorter than 15 min (*n* = 44) and longer than 3 h (*n* = 29) were excluded. Furthermore, 55 time intervals were excluded as food availability was rated below 10. Note that time intervals of one participant could not be excluded based on this criterion, as data on food availability was not available due to adding the food availability item to the EMA protocol after starting data collection. The final dataset includes 36 participants with a total of 659 time intervals.

In order to avoid estimation issues with regards to large differences in variance of the predictor and the outcome, the Level-1 predictor stress was divided by 10. To generate unbiased estimates of the within-person effect (Wang and Maxwell, 2015), the Level-1 predictors stress and state impulsivity were centred on the person-mean. The Level-2 predictor trait impulsivity was centred on the grand-mean. Data preprocessing was performed using R (version 4.2.2) (R Core Team, 2022) and RStudio (version 2022.07.2 + 576) (RStudio Team, 2022).

### 2.5 Statistical analysis

Multilevel two-part models were used for analysis, as time intervals (Level-1) were nested within individuals (Level 2) and the outcome energy intake in kcal was zero-inflated (i.e., participants consumed no energy in 50.2% [331/659] of time intervals). Multilevel two-part models treat the outcome as a combination of two parts: (1) the zero part which predicts whether an individual eats in a given time interval based on a multilevel logistic regression and (2) the continuous part which predicts how much individuals eat, if they eat in a given time interval, based on a multilevel gamma regression. Thereby they allow differentiating between stress influencing either the occurrence (zero part) or the amount of food intake (continuous part) or both, while accounting for a potential dependency between the two outcome components. Note that the multilevel logistic regression in the zero part predicts the probability *not* to eat for a given individual in a given time interval, i.e., the probability that the outcome is 0 (not 1 as commonly in logistic regressions). Estimates of the multilevel gamma regression (i.e., the continuous part of the multilevel two-part model) are modelled on the log scale, wherefore estimates in the original metric can be obtained through exponentiation. Estimates of the multilevel logistic regression (i.e., the zero part) are modelled on the logit scale, wherefore the intercept of the zero part represents the average log-odds not to eat across all participants when all predictors are 0. In order to transform the log-odds to the probability not to eat, the inverse logit function (e.g., *plogis*-function in R) can be applied. Further details on the implementation and interpretation of the multilevel two-part models used in this study can be found in Ruf et al. (2021b).

To test whether stress is generally associated with (increased) food intake in adults with ADHD, a model including the Level-1 predictor stress in both model parts was run (model 1). To examine whether trait impulsivity moderates the stress and eating relationship, six separate models including a cross-level interaction between the Level-1 predictor stress and the Level-2 predictor trait impulsivity (model 2) and the five subscales of trait impulsivity (models 2.1, 2.2., 2.3, 2.4, and 2.5) in both model parts were run. To assess whether state impulsivity moderates the stress and eating relationship, a model including an interaction between the Level-1 predictors stress and state impulsivity was run (model 3). As we expect the average probability not to eat and the average amount of energy intake to differ across individuals, all models included random intercepts in both model parts. Random slopes for the Level-1 predictors stress and state impulsivity were included to examine whether individuals differ in the effect of stress/state impulsivity.

The models were run using the R package brms (version 2.19.0) (Bürkner, 2017, 2018) and rstan (version 2.26.13) (Stan Development Team, 2022). Since brms is based on Bayesian inference, fixed effects were interpreted as significant if their credible intervals (95% CI) did not include 0. Random effects were interpreted as significant if the lower limit of the CI of their standard deviation (*SD*) was above 0.00, as nonpositive estimates for *SD* are not allowed. Model parameters were estimated based on 10,000 iterations. Defaults of all other sampling and prior parameters were used. The data and R code that support the findings of this study are available in the supplementary material of this article.

## 3 Results

Means, standard deviations, and ranges of the Level-1 and Level-2 predictors are shown in Table 2.

**Table 2:** Descriptive statistics of the Level-1 predictors stress and state impulsivity (*N* = 659) and Level-2 predictor trait impulsivity (*N* = 36).

Variable	*M*	*SD*	Range	Scale range
*Level-1*				
Stress	35.39	21.74 (overall) 16 (between)	0–99.67	0–100
State impulsivity	6.69	2.77 (overall) 1.97 (between)	4–19	4–20
*Level-2*				
Trait impulsivity (total)	30.13	5.12	18.2–40	11.8–47.2
Negative urgency	33.86	7.84	17–48	12–48
Positive urgency	32.44	9.48	14–52	14–56
(Lack of) premeditation	25.92	5.29	16–38	11–44
(Lack of) perseverance	25.22	5.23	13–35	10–40
Sensation seeking	33.19	8.83	15–48	12–48

### 3.1 General effect of stress

Model estimates of model 1 can be found in Table 3. In time intervals with an average stress level, the average probability of no energy intake is 0.51 (see intercept of the zero part; *plogis*(0.01)). Participants differ in the probability of no energy intake with an *SD* of 0.28. The 95% CI of the fixed effect of stress in the zero part includes 0 indicating that there is no significant fixed effect of stress on the probability not to eat. Furthermore, the lower limit of the 95% CI of the random effect of stress (*SD*(stress)) in the zero part indicates that the effect of stress on the probability of no energy intake does not substantively vary across participants.

**Table 3:** Model estimates of the multilevel two-part model with the Level-1 predictor stress.

	Zero part				Continuous part			
	Estimate	*SE*	95% CI		Estimate	*SE*	95% CI	
			LL	UL			LL	UL
Model 1								
*Fixed effects*								
intercept	0.01	0.10	−0.18	0.20	6.28	0.06	6.16	6.41
stress	−0.02	0.06	−0.13	0.09	−0.05	0.04	−0.12	0.03
*Random effects*								
*SD*(intercept)	0.28	0.13	0.03	0.55	0.18	0.08	0.02	0.34
*SD*(stress)	0.08	0.06	0.00	0.22	0.04	0.03	0.00	0.13

*Note.* CI = credible interval; LL = lower limit; UL = upper limit.

In time intervals with average stress in which energy intake occurred, participants consume on average 533.79 kcal (see intercept of the continuous part; exp(6.28)). The average amount of energy intake differs across individuals with an *SD* of 0.18. Stress has no fixed effect on the (log) amount of energy intake (95% CI includes 0). There is no relevant between-person variation in the effect of stress on the (log) amount of energy intake (see *SD*(stress)).

### 3.2 Moderating effect of trait impulsivity

Model estimates of model 2 are displayed in Table 4. Trait impulsivity did not moderate the stress and eating relationship in either of the two model parts and did not have a statistically meaningful main effect on the probability not to eat or on the (log) amount consumed in time intervals in which eating occurs. Beyond that, no moderating or main effect was found for the five subscales of trait impulsivity in both model parts (see model estimates in Supplement D).

**Table 4:** Model estimates of the multilevel two-part model of the moderating effect of trait impulsivity.

	Zero part				Continuous part			
	Estimate	*SE*	95% CI		Estimate	*SE*	95% CI	
			LL	UL			LL	UL
Model 2								
*Fixed effects*								
intercept	0.01	0.09	−0.17	0.20	6.29	0.06	6.17	6.41
stress	−0.02	0.06	−0.14	0.09	−0.05	0.04	−0.12	0.03
trait impulsivity	0.02	0.02	−0.01	0.06	0.02	0.01	−0.00	0.04
stress∗trait impulsivity	0.01	0.01	−0.01	0.03	−0.00	0.01	−0.01	0.01
*Random effects*								
*SD*(intercept)	0.27	0.14	0.02	0.54	0.16	0.08	0.01	0.32
*SD*(stress)	0.08	0.06	0.00	0.23	0.05	0.04	0.00	0.13

*Note.* CI = credible interval; LL = lower limit; UL = upper limit.

### 3.3 Moderating effect of state impulsivity

Model estimates of model 3 are shown in Table 5. State impulsivity did not significantly moderate the relationship between stress and the probability not to eat as well as the (log) amount consumed in time intervals in which eating occurred. No fixed effect of state impulsivity in either of the two model parts and no meaningful variation in the effect of state impulsivity as well as the interaction effect between stress and state impulsivity across participants were found.

**Table 5:** Model estimates of the multilevel two-part model of the moderating effect of state impulsivity.

	Zero part				Continuous part			
	Estimate	*SE*	95% CI		Estimate	*SE*	95% CI	
			LL	UL			LL	UL
Model 3								
*Fixed effects*								
intercept	0.00	0.10	−0.19	0.20	6.30	0.06	6.17	6.42
stress	−0.01	0.06	−0.14	0.11	−0.05	0.04	−0.13	0.04
state impulsivity	−0.02	0.05	−0.12	0.08	−0.00	0.03	−0.06	0.06
stress∗state impulsivity	0.02	0.03	−0.04	0.08	−0.01	0.02	−0.05	0.02
*Random effects*								
*SD*(intercept)	0.29	0.14	0.03	0.57	0.18	0.08	0.02	0.35
*SD*(stress)	0.08	0.06	0.00	0.24	0.05	0.03	0.00	0.13
*SD*(state impulsivity)	0.10	0.07	0.00	0.25	0.04	0.03	0.00	0.11
*SD*(stress∗state impulsivity)	0.04	0.04	0.00	0.14	0.02	0.02	0.00	0.07

*Note.* CI = credible interval; LL = lower limit; UL = upper limit.

## 4 Discussion

Adults with ADHD are at risk not only for obesity (Cortese et al., 2015) but also adverse medical outcomes, medical morbidity, and possibly premature death (Spencer et al., 2014). Stress-induced eating might represent a behavioural pathway through which ADHD psychopathology contributes to obesity and obesity-related negative health outcomes. To test whether adults with ADHD are at risk for stress-induced eating, the present study collected detailed, longitudinal data on stress and food intake in individuals with ADHD using EMA. Findings indicate that stress has no general effect on the occurrence or the amount of energy intake in adults with ADHD. Neither trait impulsivity nor state impulsivity moderates the stress and eating relationship.

The findings of the present study indicate that individuals with ADHD might not engage in eating or overeating when experiencing stress. Hence, adults with ADHD might not generally be at risk for stress-induced eating. This raises the question whether only certain individuals with ADHD are at risk for stress-induced eating as suggested by the individual-difference model (Greeno and Wing, 1994). Given that previous research indicates that particularly impulsivity symptoms of ADHD are positively associated with overeating (Kaisari et al., 2017), this study evaluated whether trait impulsivity moderates the stress and eating relationship. However, the findings of the present study indicate that trait impulsivity does not serve as a predictor for identifying individuals who (over)eat when experiencing stress. This is not surprising when considering that individual/between-person differences in the within-person effect of stress on both the occurrence and the amount of food intake were small (see lower levels of random effects of stress in model 1).

The dynamic individual-difference model (Ruf et al., 2023) proposes that not only between-person but also time-varying factors might moderate the stress and eating relationship. This suggests that individuals might not always show the same dietary response to stress, wherefore the absence of stable individual differences would not necessarily be unexpected. This model emphasizes the potential role of changing (internal and external) circumstances in shaping an individual's dietary response to stress. Based on this, it was assessed whether state impulsivity moderates the stress and eating relationship. Yet again, no moderating effect was found, indicating that the risk for stress-induced eating does not seem elevated in situations in which individuals are more impulsive. However, it is important to note that state impulsivity was rated relatively low and showed little variance in the present sample. As a consequence, the absence of a moderating effect of state impulsivity should be interpreted with caution, since this finding rests on a narrow range of state impulsivity. Future studies are needed to replicate this finding on the basis of a wider range of state impulsivity.

Given that only two thirds of participants (*n* = 24) showed elevated levels of body weight (see Table 1), one might wonder whether a positive association between stress and eating is only present in this subsample. However, this was not the case (data not shown). Furthermore, BMI and weight status (BMI <25 vs. BMI ≥25) did not moderate the relationship between stress and eating in the complete sample (data not shown).

In line with previous findings (Combs et al., 2012; Hirvikoski et al., 2009; Lackschewitz et al., 2008), we found that individuals with ADHD report higher levels of perceived stress (*M* = 35.39) compared to adults without ADHD (*M* = 18.65) (Ruf et al., 2023). Also the proportion of time intervals with elevated stress levels (>50) was considerably higher in the ADHD sample (26% of time intervals [171/659]) compared to the sample without ADHD (7% of time intervals [195/2779]) (Ruf et al., 2023). This highlights the importance of targeting stress in this population. Even though the findings of the present study did not find evidence that individuals with ADHD are at particular risk for stress-induced eating, stress is nonetheless a promising target for improving both physical and mental health in adults with ADHD, e.g., reducing internalizing symptoms (Speyer et al., 2023).

The findings of the present study must be seen in the context of some limitations. The primary limitations of this study are the relatively small sample size and the rather short EMA period, wherefore the results should be considered preliminary. In particular, the analysis of the cross-level interaction in model 2 needs replication in larger samples. Since one of the central aims of the study was to capture actual food intake (i.e., participants were asked to report all consumed foods and drinks and their amounts which allows to generate energy intake) which can be time-consuming and burdensome, an EMA period longer than three days did not seem feasible. For instance, the feasibility, usability, and validation study of the APPetite-mobile-app found that food recording latency (i.e., time between food intake and food recording) increased considerably over three days (Ruf et al., 2021a). This increase suggests that a decrease in motivation, potentially due to the high burden, could have impacted the consistent and timely reporting of food and drink consumption. Longer assessment periods seem feasible only when advanced dietary assessment methods, requiring minimal user interaction, are available for naturalistic settings. These methods may include wearable sensors that passively detect eating behaviour and accurate automatized photo-based assessments of energy content and macronutrient composition. These technological advancements could provide more reliable and less burdensome ways to gather dietary data over extended periods. The EMA period of three days might not have allowed to capture the complete spectrum of the stress and eating relationship. The short assessment period may also be related to the low levels and limited variance of state impulsivity found in the present study, as mentioned before. Another reason for the relatively low levels of state impulsivity could be systematic non-compliance. Since state impulsivity was assessed through self-reports, individuals may not reply to prompts when being impulsive, wherefore higher impulsivity ratings might be underrepresented in the data. However, since the compliance rates were high (89.6%), this bias is expected to be small. Yet, objective assessments of state impulsivity (e.g., passive detection of impulsive behaviour; Wen et al., 2021) could make a valuable addition to self-reported state impulsivity. While momentary impulsivity and stress might have caused some degree of systematic non-responding to prompts, they might have also caused non-reporting of food intake, i.e., participants might be less likely to report what they eat and drink when they are stressed or impulsive – obscuring the true relationship between stress and food intake. Tools that allow the passive detection of eating are needed to rule this out as a possible reason for not observing a relationship between stress and eating. The present study focuses solely on stress eating, as Reichenberger et al. (2018) recommend making a clearer distinction between stress and negative affect in research on stress and emotional eating. Yet, as stress and emotions are interdependent and stress is often accompanied by (negative) emotions, studying the two simultaneously could provide a holistic understanding of what influences (over)eating in daily life. Beyond that, it is important to highlight that the present EMA study does not establish temporal sequences and causal relationships and does not include a control group. Future studies should differentiate between different types of stressors (e.g., work stress, interpersonal stress) and different types of food intake (e.g., snacks vs. main meals, healthy vs. unhealthy food intake).

Despite these limitations, the present study provides first evidence in an area of high public health relevance and follows a novel, innovative, and methodologically rigorous approach. It is the first study to apply EMA to evaluate the relationship between stress and eating in adults with ADHD. EMA can make a valuable contribution to understanding stress-induced eating, as it allows to shed light on real-world microtemporal dynamics of stress and food intake. It reduces recall bias, increases ecological validity, captures within-person processes and variation over time and across settings, and thereby overcomes disadvantages of traditional approaches, such as laboratory tasks and retrospective self-reports (Shiffman et al., 2008). A further strength of the present study is the comprehensive assessment of actual food intake using a validated tool. The food record of the APPetite-mobile-app was shown to assess food intake more accurately compared to commonly used 24-h dietary recalls (Ruf et al., 2021a). Finally, the present study applied sophisticated statistical models (i.e., multilevel two-part models), which allow to study the relationship between stress and the occurrence as well as the amount of food intake, while also accounting for their dependency (Ruf et al., 2021b).

## 5 Conclusion

The present study is the first to assess stress-induced eating in adult ADHD using EMA and thereby provides first real-world evidence that adults with ADHD may not be at risk for stress-induced eating. Beyond that, no evidence was found that trait and state impulsivity moderate the stress and eating relationship in adults with ADHD. Future studies are needed to replicate these findings in larger samples and on the basis of longer EMA periods. Individuals with ADHD could greatly benefit from interventions targeting stress and overeating. Understanding and modifying stress experiences and eating behaviour in this at-risk population has the potential to considerably improve health and well-being, making it a research area of high public health significance.

## Contributors

Alea Ruf: Conceptualization, Data curation, Formal analysis, Investigation, Project administration, Writing—original draft, Writing—review and editing; Andreas B. Neubauer: Writing—review and editing; Elena D. Koch: Writing—review and editing; Ulrich Ebner-Priemer: Funding acquisition, Writing—review and editing; Andreas Reif: Conceptualization, Funding acquisition, Writing—review and editing; Silke Matura: Conceptualization, Project administration, Supervision, Writing—review and editing.

## Data availability statement

The data and R code that support the findings of this study are available in the supplementary material of this article.

## Role of the funding source

This work was supported by the European Union's Horizon 2020 Research and Innovation Program under grant agreement No 728018. The funding source has had no involvement in the study design, data collection, interpretation of the findings, or writing of this manuscript.

## Conflict of interest

The authors declare no conflict of interest.
