# Supplementary material for: Stressed! Grab a bite? Stress eating in adults with Attention-Deficit/Hyperactivity Disorder: An Ecological Momentary Assessment study
Source: Neurosci Appl. 2025 Feb 8;4:105509. doi: 10.1016/j.nsa.2025.105509 (PMC12244096; doi:10.1016/j.nsa.2025.105509)
Supplement: Multimedia component 3 [file mmc3.pdf]

Inclusion and Exclusion Criteria

PROUD study

Inclusion criteria:

- Meet DSM-5 criteria for a lifetime history of childhood onset attention deficit/hyperactivity disorder (ADHD) (DSM-5 314.00, 314.01) as well as current ADHD criteria
- 14 to 30 years of age[1]
- *For participants younger than 18 years*: written informed consent of the legal caretakers and, if possible, written assent of the participants themselves
- *For participants of at least 18 years of age:* Written informed consent of the participant
- Stable treatment as usual comprising pharmacotherapy, group-based or individual cognitive-behavioral therapy (not including elements of bright light therapy or exercise interventions)
- Normal or corrected vision
- Understand, read, write, and speak the language of the study site fluently[2]
- Ability to regularly and reliably attend appointments

Exclusion criteria:

- IQ above 75 (measured by WAIS-IV or WISC-IV vocabulary and matrix reasoning subtests)
- Severe psychiatric disorder other than the co-morbid conditions explicitly studied with necessary additional psychopharmacotherapy or psychiatric intervention involving day-care/inpatient treatment at start of study, especially a diagnosis of bipolar disorder, schizophrenia, autism spectrum disorder, schizoaffective disorder or organic psychiatric disorder (current or lifetime)
- Severe medical or neurological condition interfering with the intervention
- Severe medical or neurological condition not allowing bright light therapy or exercise intervention
- Use of antipsychotic or anti-epileptic medication, photo-sensitizing medication (e.g. lithium, St. John's Wort)
- Substance use disorder (DSM-5) or dependency (DSM-5)

---

[1] Only PROUD participants of at least 18 years were recruited for the APPetite study.

[2] Only PROUD participants who completed the study in Frankfurt (Germany) were recruited for the APPetite study.

- History of epilepsy
- Acute suicidal ideation
- Pregnancy
- Participant is related to the investigator or study staff
- Participation in other clinical trials and observation period of competing trials (participation in other studies is permitted if the respective study is a no-medication or psychotherapy trial and if its aims do not interfere with the aims of the PROUD study)

See Mayer et al. (2018) for more details.

BipoLife-A1 study

Inclusion criteria:

- Age: 15 to 35 years[3]
- In- or outpatients with a clinically confirmed ADHD diagnosis

Exclusion criteria:

- Diagnosis of bipolar disorder, schizoaffective disorder, or schizophrenia
- Diagnosis of anxiety, obsessive–compulsive, or substance dependence disorder fully explaining the symptomatology
- Limited ability to comprehend the study
- Implied expressed negative declaration of intent to participate in the study by a minor
- Acute suicidality

See Pfennig et al. (2020) for more details.

PROBIA study

Inclusion criteria:

- Both males and females aged 18–65 years
- High level of multidimensional impulsivity based on the Clinical Global Impression–Severity Scale score ≥ 4 and the Affective Reactivity Index ≥ 5
- DSM-5 criteria for ADHD and/or borderline personality disorder confirmed by a structured diagnostic interview (ADHD: Diagnostic Interview for Adult ADHD [DIVA 2.0]; BPD: Structured Clinical Interview for DSM-IV [SCID-II])[4]

---

[3] Only participants of the BipoLife-A1 study who were at least 18 years of age were recruited for the APPetite study.

[4] Only PROBIA participants who met at least the criteria for ADHD were recruited for the APPetite study.

- Deemed reliable and compliant with the protocol by the investigator
- Ability to speak and comprehend the native language of the country in which the assessments take place[5]
- Informed consent

Exclusion criteria:

- Use of antibiotics in the last 6 weeks
- Current intake of probiotics
- Major psychiatric disorder with psychotic symptoms or other major psychiatric conditions requiring hospitalization (e.g., significant mood disorders)
- Neurological disorders involving central functions (e.g., epilepsy, multiple sclerosis, narcolepsy)
- IQ above 70 (measured by WAIS)
- Major physical illnesses of the cardiovascular, endocrine, pulmonal, immune, or gastrointestinal system or undergoing immunosuppression
- History of/present clinically relevant somatic acute or chronic disorders that might confound the results of tolerability/safety assessments or prohibit the patient from completing the study or would not be in the best interest of the patient
- Allergy, hypersensitivity, or intolerance to any of the ingredients of the intervention
- Use of another investigational product or participating in a clinical study in the last 30 days

See Arteaga-Henríquez et al. (2020) for more details.

[5] Only PROBIA participants who completed the study in Frankfurt (Germany) were recruited for the APPetite study.

References

Arteaga-Henríquez, G., Rosales-Ortiz, S. K., Arias-Vásquez, A., Bitter, I., Ginsberg, Y., Ibañez-Jimenez, P., Kilencz, T., Lavebratt, C., Matura, S., Reif, A., Rethelyi, J., Richarte, V., Rommelse, N., Siegl, A., & Ramos-Quiroga, J. A. (2020). Treating impulsivity with probiotics in adults (PROBIA): study protocol of a multicenter, double-blind, randomized, placebo-controlled trial. *Trials*, *21*(1), 161. https://doi.org/10.1186/s13063-019-4040-x

Mayer, J. S., Hees, K., Medda, J., Grimm, O., Asherson, P., Bellina, M., Colla, M., Ibáñez, P., Koch, E., Martinez-Nicolas, A., Muntaner-Mas, A., Rommel, A., Rommelse, N., de Ruiter, S., Ebner-Priemer, U. W., Kieser, M., Ortega, F. B., Thome, J., Buitelaar, J. K., … Freitag, C. M. (2018). Bright light therapy versus physical exercise to prevent co-morbid depression and obesity in adolescents and young adults with attention-deficit / hyperactivity disorder: study protocol for a randomized controlled trial. *Trials*, *19*(1), 140. https://doi.org/10.1186/s13063-017-2426-1

Pfennig, A., Leopold, K., Martini, J., Boehme, A., Lambert, M., Stamm, T., Bermpohl, F., Reif, A., Kittel-Schneider, S., Juckel, G., Fallgatter, A. J., Kircher, T., Jansen, A., Pfeiffer, S., Berndt, C., Rottmann-Wolf, M., Sauer, C., Ritter, P., Correll, C. U., … Bauer, M. (2020). Improving early recognition and intervention in people at increased risk for the development of bipolar disorder: study protocol of a prospective-longitudinal, naturalistic cohort study (Early-BipoLife). *International Journal of Bipolar Disorders*, *8*(1). https://doi.org/10.1186/s40345-020-00183-4
