# Supplementary material for: Stressed! Grab a bite? Stress eating in adults with Attention-Deficit/Hyperactivity Disorder: An Ecological Momentary Assessment study
Source: Neurosci Appl. 2025 Feb 8;4:105509. doi: 10.1016/j.nsa.2025.105509 (PMC12244096; doi:10.1016/j.nsa.2025.105509)
Supplement: Multimedia component 4 [file mmc4.pdf]

Table D.1

Model Estimates of the Multilevel Two-Part Model of the Moderating Effect of Negative Urgency

	Zero part				Continuous part			
	Estimate	*SE*	95% CI		Estimate	*SE*	95% CI	
			LL	UL			LL	UL
				model 2.1				
Fixed effects								
intercept	0.02	0.09	–0.17	0.20	6.29	0.06	6.17	6.41
stress	–0.03	0.06	–0.14	0.09	–0.05	0.04	–0.12	0.03
negative urgency	0.01	0.01	–0.01	0.04	0.01	0.01	–0.00	0.03
stress*negative urgency	0.01	0.01	–0.01	0.02	–0.00	0.01	–0.01	0.01
Random effects								
SD(intercept)	0.26	0.14	0.02	0.54	0.16	0.08	0.01	0.32
SD(stress)	0.08	0.06	0.00	0.23	0.04	0.03	0.00	0.13

Note. CI = credible interval; LL = lower limit; UL = upper limit.

Table D.2

Model Estimates of the Multilevel Two-Part Model of the Moderating Effect of Positive Urgency

	Zero part				Continuous part			
	Estimate	*SE*	95% CI		Estimate	*SE*	95% CI	
			LL	UL			LL	UL
	model 2.2							
Fixed effects								
intercept	0.02	0.09	–0.17	0.21	6.29	0.06	6.17	6.41
stress	–0.02	0.06	–0.14	0.09	–0.05	0.04	–0.12	0.03
positive urgency	0.01	0.01	–0.01	0.03	0.01	0.01	–0.00	0.02
stress*positive urgency	0.00	0.01	–0.01	0.01	–0.00	0.00	–0.01	0.01
Random effects								
SD(intercept)	0.26	0.14	0.02	0.54	0.16	0.08	0.02	0.33
SD(stress)	0.08	0.06	0.00	0.23	0.05	0.04	0.00	0.14

Note. CI = credible interval; LL = lower limit; UL = upper limit.

Table D.3

Model Estimates of the Multilevel Two-Part Model of the Moderating Effect of (Lack of) Premeditation

	Zero part				Continuous part			
	Estimate	*SE*	95% CI		Estimate	*SE*	95% CI	
			LL	UL			LL	UL
				model 2.3				
Fixed effects								
intercept	0.01	0.10	–0.18	0.20	6.29	0.06	6.17	6.42
stress	–0.02	0.06	–0.14	0.09	–0.05	0.04	–0.13	0.03
(Lack of) Premeditation	–0.01	0.02	–0.05	0.02	–0.01	0.01	–0.03	0.02
stress*(Lack of) Premeditation	0.01	0.01	–0.01	0.04	0.00	0.01	–0.01	0.02
Random effects								
SD(intercept)	0.29	0.13	0.03	0.56	0.19	0.08	0.02	0.35
SD(stress)	0.07	0.06	0.00	0.22	0.04	0.03	0.00	0.13

Note. CI = credible interval; LL = lower limit; UL = upper limit.

Table D.4

Model Estimates of the Multilevel Two-Part Model of the Moderating Effect of (Lack of) Perseverance

	Zero part				Continuous part			
	Estimate	*SE*	95% CI		Estimate	*SE*	95% CI	
			LL	UL			LL	UL
				model 2.4				
Fixed effects								
intercept	0.01	0.09	–0.17	0.20	6.29	0.06	6.17	6.41
stress	–0.02	0.06	–0.15	0.11	–0.05	0.04	–0.14	0.03
(Lack of) Perseverance	0.02	0.02	–0.01	0.06	0.01	0.01	–0.01	0.03
stress*(Lack of) Perseverance	0.00	0.01	–0.02	0.02	0.00	0.01	–0.01	0.02
Random effects								
SD(intercept)	0.27	0.14	0.02	0.54	0.18	0.08	0.02	0.34
SD(stress)	0.08	0.06	0.00	0.23	0.05	0.03	0.00	0.13

Note. CI = credible interval; LL = lower limit; UL = upper limit.

Table D.5

Model Estimates of the Multilevel Two-Part Model of the Moderating Effect of Sensation Seeking

	Zero part				Continuous part			
	Estimate	*SE*	95% CI		Estimate	*SE*	95% CI	
			LL	UL			LL	UL
	model 2.5							
Fixed effects								
intercept	0.01	0.10	–0.18	0.20	6.29	0.06	6.16	6.41
stress	–0.02	0.06	–0.13	0.10	–0.05	0.04	–0.12	0.03
Sensation Seeking	0.01	0.01	–0.01	0.03	0.01	0.01	–0.01	0.02
stress*Sensation Seeking	–0.00	0.01	–0.01	0.01	0.00	0.00	–0.01	0.01
Random effects								
SD(intercept)	0.29	0.14	0.03	0.56	0.17	0.08	0.02	0.34
SD(stress)	0.08	0.06	0.00	0.23	0.05	0.04	0.00	0.13

Note. CI = credible interval; LL = lower limit; UL = upper limit.
